# Preclinical efficacy of combination therapy with allogeneic induced pluripotent stem cell-derived invariant natural killer T and α-galactosylceramide-pulsed antigen-presenting cells

**DOI:** 10.1186/s13287-026-04994-7

**Published:** 2026-03-29

**Authors:** Takahiro Aoki, Midori Kobayashi, Momoko Okoshi, Munechika Yamaguchi, Hiroko Okura, Satoko Sasaki, Yoshie Sasako, Sachiko Kira, Yun-Hsuan Chang, Nayuta Yakushiji-Kaminatsui, Jafar Sharif, Masashi Matsuda, Masahiro Kiuchi, Kiyoshi Hirahara, Motoko Y. Kimura, Shinichiro Motohashi, Haruhiko Koseki

**Affiliations:** 1 Laboratory for Developmental Genetics, RIKEN Center for Integrative Medical Sciences, 1-7-29 Suehiro-cho, Tsurumi-ku, Yokohama, Kanagawa 230-0045 Japan; 2 Department of Medical Immunology, Graduate School of Medicine, Chiba University, 1-8-1 Inohana, Chuo-ku, Chiba, Chiba 260-8670 Japan; 3 Department of Cellular and Molecular Medicine, Graduate School of Medicine, Chiba University, Chuo-ku, Chiba, 260-8670 Japan; 4 Department of Immunology, Graduate School of Medicine, Chiba University, Chuo-ku, Chiba, 260-8670 Japan; 5 Department of Experimental Immunology, Graduate School of Medicine, Chiba University, Chuo-ku, Chiba, 260-8670 Japan

**Keywords:** Invariant natural killer T cells, iPS cells, α-galactosylceramide, Acquired immunity, Preclinical study, Patient-derived xenograft model, Single-cell analysis

## Abstract

Invariant natural killer T (iNKT) cells, upon activation, exhibit antitumor roles by bridging innate and acquired immunity. To overcome the challenges in producing iNKT cells from patients with cancer, we previously developed allogeneic human induced pluripotent stem cell-derived iNKT (iPSC-iNKT) cells. However, the activation of iPSC-iNKT cells by glycolipid ligands remains a critical step for iNKT cell-mediated cancer therapy. To show the effect of iPSC-iNKT cell-mediated antitumor immunity, in this preclinical study, by taking advantage of a human immune cell-transplanted patient-derived xenograft model using human IL-7/15 knock-in NSG mice, we demonstrate that a combination of iPSC-iNKT cells and α-galactosylceramide-pulsed antigen-presenting cells (αGalCer/APC) induces robust antitumor effects. Single-cell analysis of tumor-infiltrating lymphocytes revealed that this combination therapy uniquely expanded tumor-reactive memory-phenotype CD4 and CD8 T cells. Taken together, upon activation by αGalCer/APC, iPSC-iNKT cells are capable of effectively inducing antitumor T cell immunity, making them a promising tool for generating personalized antitumor T cell immunity.

**Supplementary Information:**

The online version contains supplementary material available at 10.1186/s13287-026-04994-7.

## Background

Invariant natural killer T (iNKT) cells are a distinct subset of innate-like T cells characterized by an invariant T cell receptor (TCR) [1]. This TCR is encoded by Vα14 Jα18 and Vα24 Jα18, in mice and humans, respectively [2–5]. Unlike conventional αβ T cells that recognize peptide antigens presented by polymorphic MHC class I or II molecules, iNKT cells recognize glycolipid antigens presented by CD1d, a monomorphic MHC class I-like molecule [6]. Identification of α-galactosylceramide (αGalCer) as a potent activator of iNKT cells marked a significant breakthrough in understanding iNKT cell function [7, 8]. iNKT cells rapidly produce large amounts of cytokines and chemokines upon activation, modulating other immune cells such as dendritic cells (DCs), NK cells, and T cells [8, 9]. Therefore, harnessing the ability of iNKT cells could be an attractive avenue for immunotherapy.

Antitumor potential of iNKT cells is mediated by tumor immunity through various mechanisms. Activated iNKT cells directly kill various cancer cell lines and indirectly enhance tumor cell elimination by stimulating other immune cells [10–12]. iNKT cells, upon activation by αGalCer, help DC maturation and thereby amplify innate and adaptive immune responses [9, 13, 14]. For example, combined administration of OVA and αGalCer to mice led to DC maturation in an iNKT cell-dependent manner and induced OVA-specific T-cell immunity [15]. In addition to the roles of iNKT cells and αGalCer in cancer immunity, viral stimulation with αGalCer also protects against infection in vivo by inducing memory cytotoxic T cells [16].

Another hallmark of the therapeutic potential of iNKT cells is their universality. Due to the monomorphic nature of CD1d, αGalCer can activate immune cells in a widespread manner by engaging iNKT cells in different individuals, bypassing the highly polymorphic nature of classical MHC molecules. In addition, allogeneic iNKT cells do not cause graft-versus-host disease because iNKT cells are not HLA-restricted, thereby opening a wide range of possibilities for the use of iNKT cells in clinical applications.

The success of iNKT cell-targeted immunotherapy in mouse tumor models has led to clinical trials at Chiba University Hospital for advanced lung and head and neck cancers [17–20]. Immunological monitoring revealed that higher tumor-infiltrating iNKT cell counts correlated with improved tumor control; however, reliable sourcing of sufficient autologous iNKT cells from cancer patients emerged as a critical problem [17, 20]. To circumvent this problem, we surmised that iPSC-derived iNKT (iPSC-iNKT) cells might be a promising source of functionally competent iNKT cells [21, 22]. We then developed human iPSC-iNKT cells [23] and conducted the first-in-human phase I clinical trial using allogeneic iPSC-iNKT cell monotherapy for recurrent head and neck cancer to test its safety and feasibility (jCRT2033200116). Because iNKT cell activation improves the antitumor effect in clinical trials [20, 24], we subsequently planned a combination therapy with iPSC-iNKT cells and αGalCer/APC to trigger potent antitumor immunity.

However, to implement this combination therapy, it is necessary to confirm that human iPSC-iNKT cells retain the adjuvant function as observed previously with murine iPSC-iNKT cells [25]. Furthermore, information available on the adjuvant activity of human NKT cells remains limited. To address this issue, in the present preclinical study, we analyze the influence of activated human iPSC-iNKT cells on T cell phenotypes and TCR repertoires at the single-cell level using a human immune cell-transplanted patient-derived xenograft model.

## Results

### Efficiency of iPSC-iNKT cell and αGalCer-pulsed APC combined therapy

**Fig. 1:**
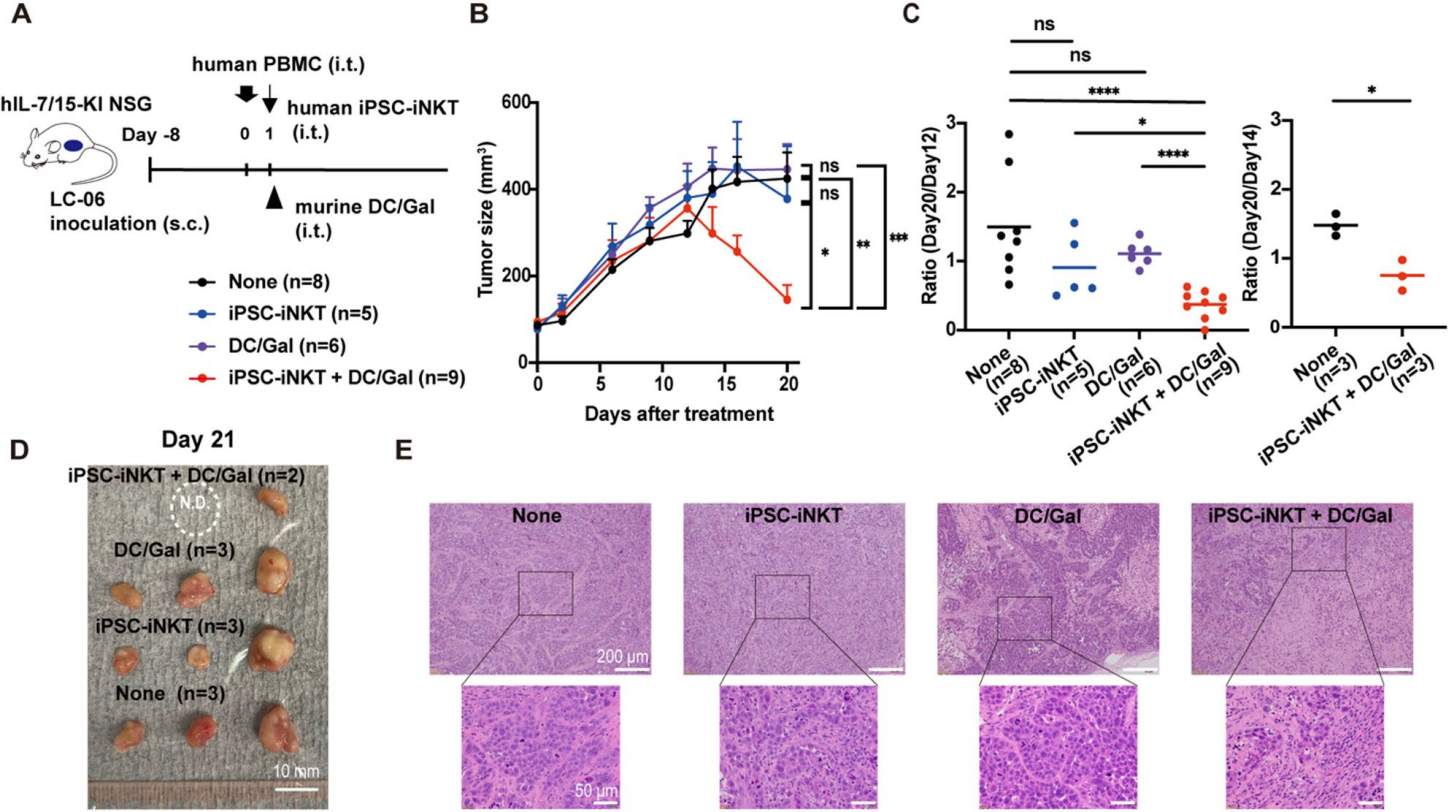
Antitumor effect of iPSC-iNKT cell therapy in combination with αGalCer/APC. **A** Experimental scheme of the PBMC-transplanted PDX model. A patient-derived lung cancer cells, LC06, and human PBMCs were transplanted into human IL-7/15 knock-in NSG mice at day − 8 and 0, respectively. These tumor-bearing mice were further treated by intratumor injection (i.t.) of iPSC-iNKT cells (iPSC-iNKT: *n* = 5), murine DC/Gal (DC/Gal: *n* = 6), both (iPSC-iNKT + DC/Gal: *n* = 9) or none (None: *n* = 8) on day 1. Tumor size was measured until day 20. s.c., subcutaneous injection; i.t., intratumor injection. **B** The tumor size in each treatment group. Data represent mean ± SEM. ***P < 0.001; **P < 0.01; *P < 0.05; ns, not significant (unpaired t-test). See also Fig. S1A. These results were obtained from four independent experimental groups. **C** The tumor size ratio (size at day 20 divided by the peak tumor size on day 12 or day 14) in experiments using PBMCs from two donors (left; donor 1, right; donor 2). Bars represent the mean. ****P < 0.0001; *P < 0.05; ns, not significant (unpaired t-test). **D** Representative images of tumors on day 21. N.D., no detectable tumor. These materials were obtained from two independent experimental groups from those shown in (A)-(C) (iPSC-iNKT + DC/Gal: *n* = 2, DC/Gal: *n* = 3, iPSC-iNKT: *n* = 3, and none: *n* = 3). **E** Representative images of tumor sections shown in (**D**) stained by hematoxylin and eosin

To evaluate the antitumor effect of iPSC-iNKT cell therapy and T cell immunity mediated by iPSC-iNKT cells, we established a human peripheral blood mononuclear cell (PBMC)-transplanted patient-derived xenograft (PDX) model in mice (Fig. 1A). Combination therapy with iPSC-iNKT cells and DC/Gal suppressed tumor size compared with untreated, only DC/Gal-treated, or only iPSC-iNKT cell-treated mice (Fig. 1B-D and S1A). Interestingly, tumor reduction began approximately 12 days after treatment (Fig. 1B), highlighting the time frame required to develop antitumor adaptive immunity. Owing to the variation in the maximum LC-06 tumor size, we determined the antitumor efficacy by dividing the tumor size on day 20 by the peak size on day 12 or 14 (Fig. 1C). The tumor size ratio showed that DC/Gal combined with iPSC-iNKT cells significantly suppressed tumor volume compared with the other treatment groups. Importantly, a reduction in tumor size was not noted in the absence of transplanted human PBMCs, even when DC/Gal was administered together with iPSC-iNKT cells (Fig. S1B, C), indicating that the observed cytotoxicity did not completely depend on the direct effects of iPSC-iNKT cells.

Hematoxylin and eosin staining of the tumors remaining on day 21 confirmed the immune cell-derived tumor regression (Fig. 1E). While dense sheets or nests of tumor cells were evident with few immune cells in the tumor in which PBMCs alone were injected, residual tumor cells were scattered in the tumor receiving the combination therapy. Although treatment with iPSC-iNKT or DC/Gal alone resulted in some level of tumor destruction, this effect was markedly weaker compared with those following the combination therapy.

### Kinetics of tumor-infiltrating lymphocytes

To identify the effector cells exhibiting the antitumor effect, we examined tumor-infiltrating human lymphocytes (TILs) during days 7 to 21 using flow cytometry (Fig. S1D). While iNKT cells, including iPSC-iNKT cells, and NK cells gradually disappeared, T cells became predominant by day 14. We performed scRNA-seq with human CD45^+^ TILs on day 14 as the antitumor effect of this therapy became apparent only after day 12 (Fig. 1B) and confirmed that the predominant TILs were T cells (Fig. S1E). We, therefore, focused on T cells to determine the precise mechanisms responsible for the antitumor effects.

**Fig. 2:**
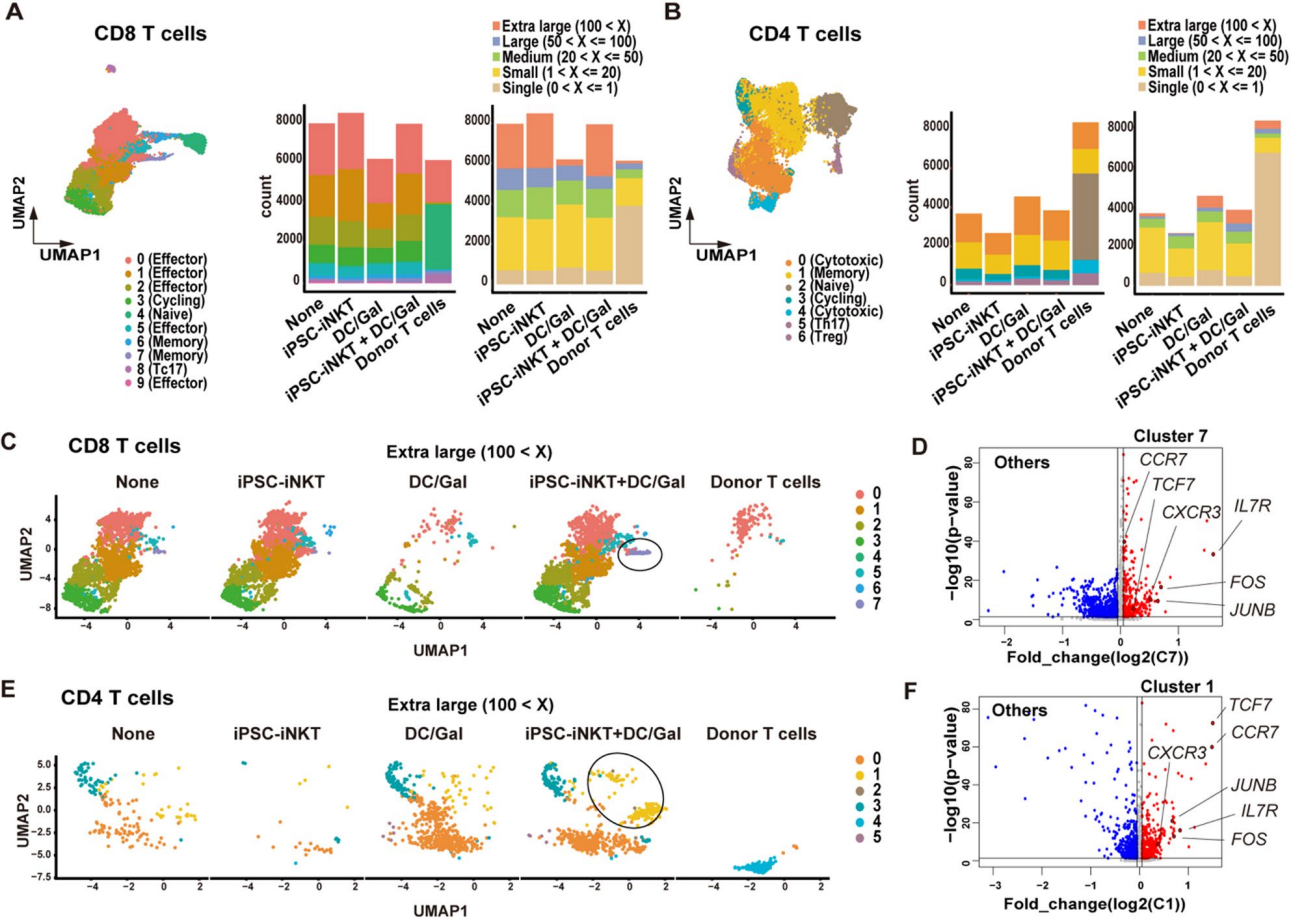
Comparison of tumor-infiltrating lymphocytes on day 14 in each treatment group. **A** UMAP of CD8 T cell clusters and bar plots split by treatment group. On the UMAP, CD8 T cells are stratified into 10 clusters (cluster 0–9) as annotated by feature gene expression shown in the left panel of Fig. S2A. The left bar plot shows clusters identified by RNA expression, whereas the right plot shows clusters identified by TCR clone size. Each treatment group contains TILs from two mice. Donor T cells represent preinjected T cells in PBMCs. **B** UMAP of CD4 T cell clusters and bar plots split by treatment group. On the UMAP, CD4 T cells are stratified into 7 clusters (cluster 0–6) as annotated by feature gene expression shown in the left panel of Fig. S2B. The left bar plot shows clusters identified by RNA expression, whereas the right plot shows clusters identified by TCR clone size. Each treatment group contains TILs from two mice. Donor T cells represent preinjected T cells in PBMCs. **C** UMAP of CD8 T cells with extra-large TCR clone size split by treatment group and preinjected donor T cells. The cluster 7 is indicated by a circle. See also Fig. S1F. **D** Volcano plot of cluster 7 and other clusters in CD8 T cells with extra-large TCR clone size. **E** UMAP of CD4 T cells with extra-large TCR clone size split by treatment group and preinjected donor T cells. The circled region indicates cluster 1. See also Fig. S1G. **F** Volcano plot of cluster 1 and other clusters in CD4 T cells with extra-large TCR clone size

To identify T cell characteristics among treatment groups, we performed single-cell RNA sequencing (scRNA-seq) analysis and clustered CD8 and CD4 T cells by their RNA expression levels and TCR clone sizes. Although we detected no significant differences in T cell phenotypes among the treatment groups, the population of T cells with extra-large TCR clone sizes and their phenotypes were significantly different (Fig. 2A, B). In the CD8 T cells with extra-large TCR clone sizes, cluster 7 was unique in the combined therapy group (Fig. 2C, circled and Fig. S1F). This cluster was characterized by the expression of marker genes for memory functions, such as *TCF7*,* CCR7* and *IL7R*, and marker genes for immune activation, such as *FOS* and *JUNB* (Fig. 2D). In CD4 T cells with extra-large TCR clone sizes in the combined therapy group, cluster 1 showed upregulation of marker genes for memory functions and immune activation (Fig. 2E, F). Based on these findings, we concluded that the combination therapy could specifically induce activating and proliferative memory-phenotype T cells. Given that only the combination showed an antitumor effect, we surmised that this memory-phenotype population is important to mediate the antitumor effect.

### Antitumor effect of the memory-phenotype T cells induced by iPSC-iNKT cell therapy

**Fig. 3:**
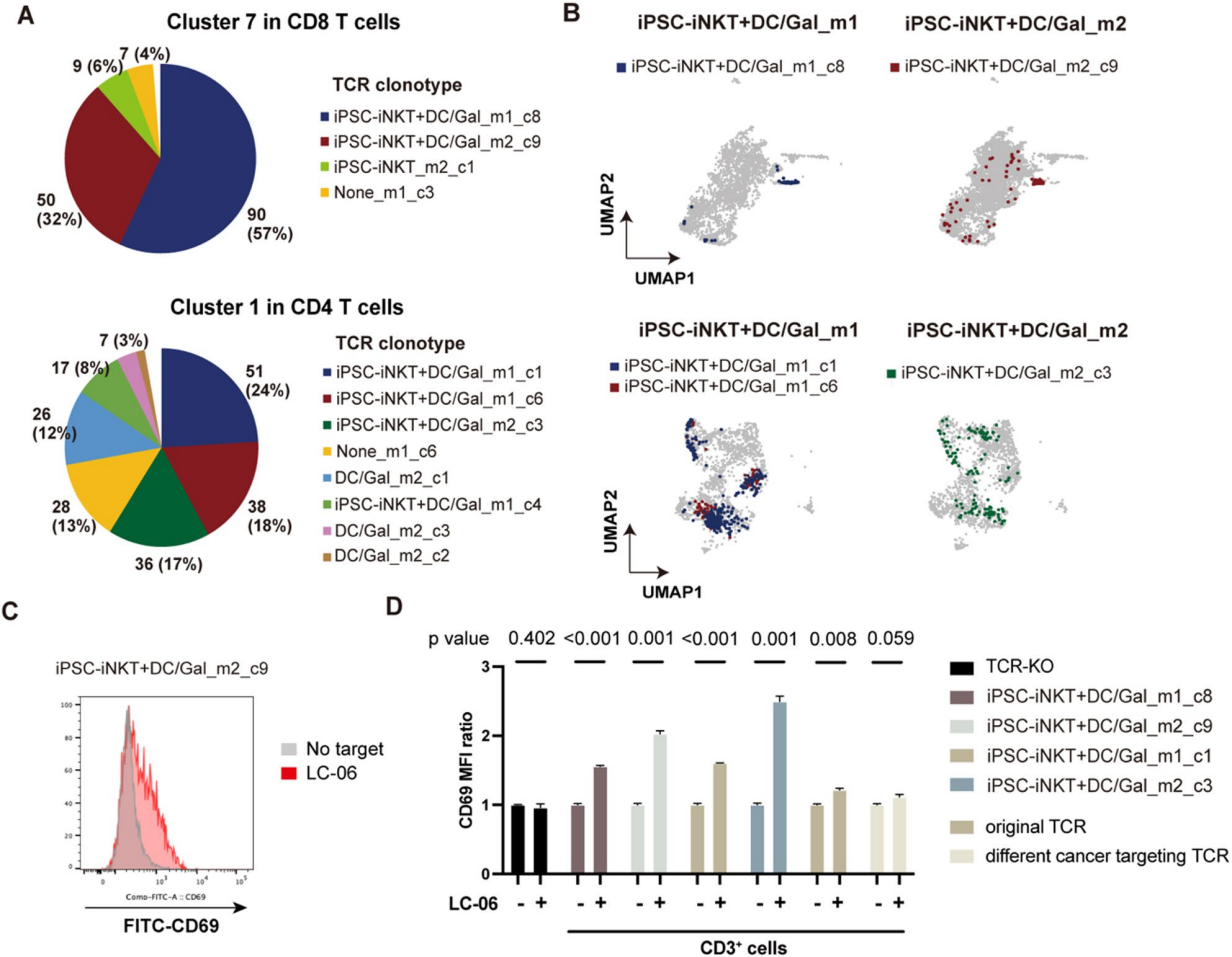
Tumor reactivity of the expanded memory-phenotype T cells. **A** The distribution of clonotypes in cluster 7 in CD8 T cells (top) and clonotypes in cluster 1 in CD4 T cells (bottom). The numbers represent the cell counts of each clonotype. Clonotypes are named by treatment groups, mouse numbers, and size ranks. For example, iPSC-iNKT + DC/Gal_m1_c8 represents the eighth largest clonotype in mouse 1 treated with iPSC-iNKT cells and DC/Gal. See also Table S1. **B** Distribution of cells with highly expanded clonotypes on CD8 T cell UMAP (top) and clonotypes on CD4 T cell UMAP (bottom). Note that expanded CD8 T cell clones and CD4 T cell clones were enriched in cluster 7 and cluster 1, respectively. Results in whole cohorts are shown in Fig. S2C and S2D. **C** Flow cytometry plots of CD69 expression in TCRα/β-KO Jurkat cells, in which TCR expressed in clonally expanded CD8 T cell clone (clonotype: iPSC-iNKT + DC/Gal_m2_c9) was reconstituted upon co-culture with (red) or without (grey) LC-06 cells. **D** CD69 expression on TCRα/β-KO Jurkat cells expressing TCRs of indicated clonotypes upon 22 h co-culture with (+) or without (–) LC-06 cells. Original Jurkat cells and TCRα/β-KO Jurkat cells expressing TCR specific for REH leukemia cell line were used as controls. Data are shown as the mean ± SD. Unpaired t-test was performed. Data are representative of two independent experiments

To evaluate the tumor reactivity of memory-phenotype T cells, we determined the TCR composition of these cells. Clonotypes of cluster 7 in CD8 T cells and cluster 1 in CD4 T cells were oligoclonal (Fig. 3A). TCRs identified as memory-phenotype were also distributed in the effector-phenotype clusters (Fig. 3B and S2A, B). We did not identify these clonotypes in other treatment groups (Fig. S2C, D). To confirm the tumor reactivity of these clonotypes, we transfected their TCRs into TCRα/β-KO Jurkat cells and assessed T cell activation by measuring CD69 expression. TCR-transfected Jurkat cells showed an increase in the level of CD69 expression after co-culture with LC06 cells (Fig. 3C, D). In contrast, LC06-induced activation was limited in TCRα/β-KO Jurkat cells expressing REH (a leukemia cell line)-reactive TCR or original Jurkat cells (Fig. 3C, D). These findings, therefore, indicated that memory-phenotype T cells were reactive to LC06 cells. However, this conclusion may be limited, as we tested only one TCR from both the CD8 and CD4 subsets.

**Fig. 4:**
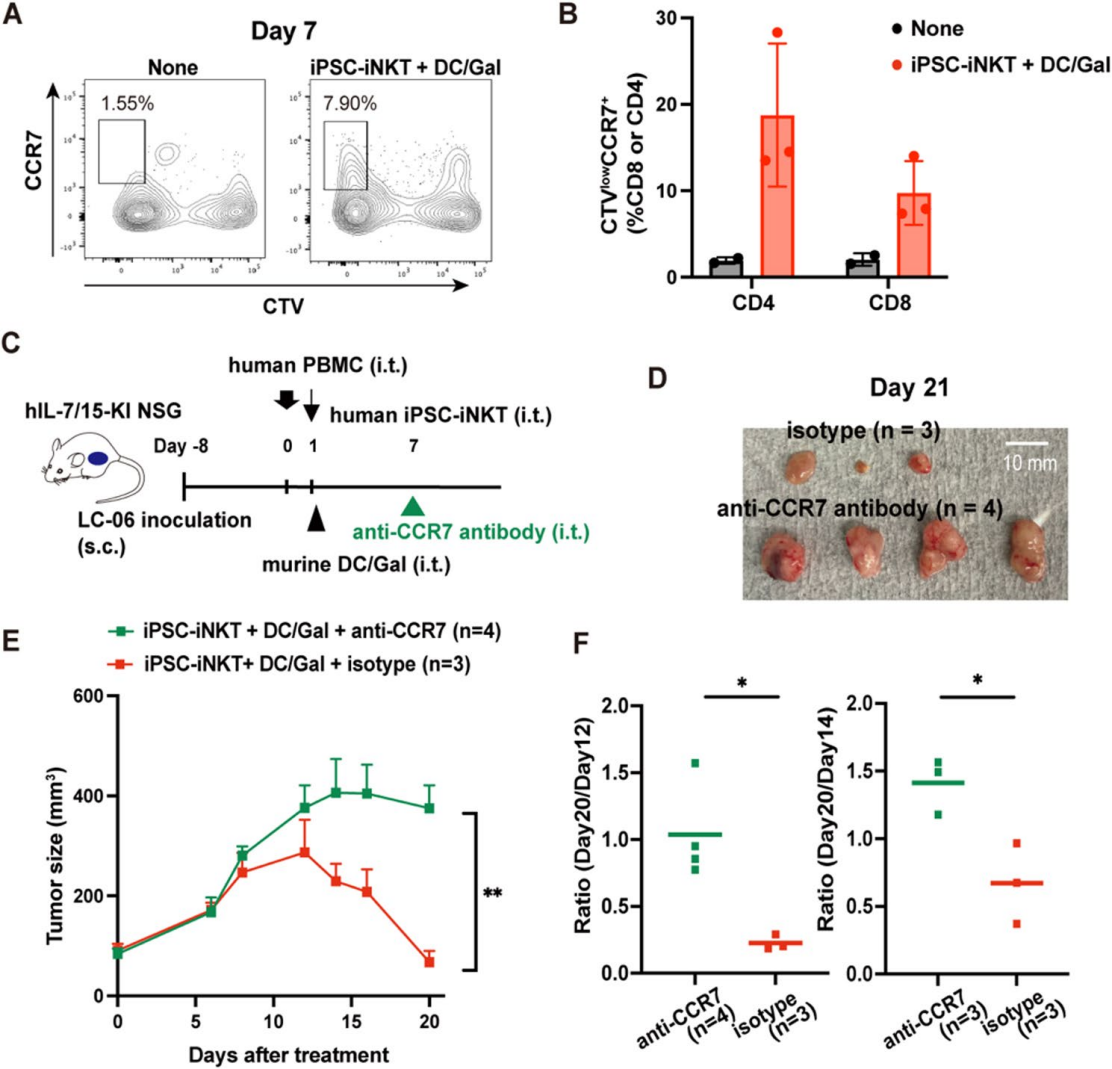
Antitumor effect of the expanded memory-phenotype T cells. **A** Representative flow cytometry plots of day 7 tumor-infiltrating CD8 T cells after treatment to identify expanded memory-phenotype T cells. **B** Summary of the expanded memory-phenotype T cells in day 7 tumor-infiltrating CD4 and CD8 T cells identified by flow cytometry. Data represent mean ± SD. Each dot represents one tumor. **C** Experimental scheme of PBMC-transplanted PDX model. We injected an anti-human CCR7 antibody into the tumor on day 7 to deplete CCR7^+^ memory-phenotype T cells. s.c., subcutaneous injection; i.t., intratumor injection. **D** Representative images of tumors on day 21 treated with or without anti-CCR7 antibody. **E** Tumor size in each treatment group (anti-CCR7: *n* = 4 and isotype: *n* = 3). Data represent mean ± SEM. **P < 0.01; ns, not significant (unpaired t-test). Data are representative of two independent experiments. **F** Tumor size ratio (size at day 20 divided by the peak tumor size on day 12 or day 14) in experiments using PBMCs from two donors (left: donor 1 and right: donor 2). Bars represent the mean. *P < 0.05 (unpaired t-test).

We examined whether these memory-phenotype T cells induced by the combination therapy were essential for the observed antitumor effect. We found that CCR7, which is a marker for memory-phenotype T cells, was indeed upregulated in cluster 7 in CD8 T cells and cluster 1 in CD4 T cells by the combination therapy. We investigated whether CCR7-positive (CCR7^+^) T cells were induced via oligoclonal expansion of their precursors using CellTrace Violet (CTV)-labeled PBMCs. This analysis confirmed that CTV_low_/CCR7^+^ population was induced by the combination therapy, representing proliferation-dependent emergence of memory-phenotype CD4 and CD8 T cells (Fig. 4A, B). To examine the antitumor roles of CCR7^+^ T cells, we depleted these cells by an anti-human CCR7 antibody [26] in the PDX model (Fig. 4C). As CTV_low_/CCR7^+^ T cell propagation took place 7 days after the combination therapy, we injected anti-human CCR7 into tumors on day 7 (Fig. 4A, C). As expected, CCR7^+^ T cells were efficiently depleted by anti-CCR7 administration (Fig. S2E), causing attenuation of antitumor effect (Figs. 4D, E, F). In summary, we reveal that the combination therapy induces tumor-reactive memory-phenotype T cells and exerts an antitumor effect.

## Discussion

In the present study, we found that incorporation of αGalCer/APC significantly enhanced the antitumor efficacy of iPSC-iNKT cells in a human immune cell-transplanted PDX model. The absence of tumor regression with PBMCs alone (Fig. 1B), or with combination therapy without human PBMC transplantation (Fig. S1B, C), indicated that the antitumor effect mediated by the combination therapy arises from acquired T cell immunity conferred by activated iPSC-iNKT cells. By analyzing TILs, we identified an expansion of memory-phenotype T cells capable of responding to LC06, which was exclusively observed in the combined therapy cohort. While human DCs may be more potent at priming and activating tumor-reactive T cells upon receiving signals from activated iPSC-iNKT cells, we found that murine αGalCer/APC can exert therapeutic effects in our model. This is because human NKT cells can recognize αGalCer presented by CD1d across mammalian species [27]. However, given the strong species restriction of MHC class I, murine APCs are unlikely to directly prime human tumor antigen–specific CTLs in our experimental setting. Therefore, the role of murine αGalCer/APC should be limited in activating iPSC-iNKT cells. Moreover, canonical αGalCer-driven antigen-specific CTL priming requires co-presentation of αGalCer on CD1d and tumor-derived peptides on MHC class I by the same APC [28], which is improbable in this xenogeneic setting. Thus, the observed efficacy may be mediated by alternative mechanisms of adjuvant-like effects of iNKT cells [29] and/or allogeneic-like immune reactivity.

In comparison to our previous study using murine iPSC-iNKT cells [25], in the present study, we used allogeneic iNKT cells derived from iPS cells from a healthy donor. We revealed that allogeneic iPSC-iNKT cells could still induce antitumor T cell immunity because iNKT cells activate other immune cells in an HLA-independent manner. This evidence motivated us to apply a combination therapy with allogeneic iPSC-iNKT cells and αGalCer/APC in a clinical trial for head and neck cancer (jRCTa030220741).

Of note, while chimeric antigen receptor (CAR) T- or iNKT cell therapies have been previously developed from autologous cells [30, 31], there is a trend to use allogeneic cells because manufacturing autologous T cells is time-consuming and expensive [32–34]. Compared with T cell-based methods, our iNKT cell-based method provides several clear advantages. First, iNKT cells do not lead to graft-versus-host disease [35]. Second, host immunity activated through the iNKT cell adjuvant activity lasts beyond the rejection of the allogeneic cells [36], hence overcoming the lack of durability of the antitumor effects dependent on CAR-T cell persistence [37]. Furthermore, our iPSC-iNKT cells can be supplied stably on an industrial scale, which will pave the way for future clinical application of iNKT-cell therapy.

We also observed a notable expansion of CD4 T cells with CD8 T cells during the combination therapy. This finding is consistent with prior reports demonstrating that activation of iNKT cells enhances CD4 and CD8 responses [15, 38]. Concomitantly, both CD4 and CD8 T cells are essential for iNKT cell-targeted therapies to effectively induce antitumor T cell immunity [14]. In recent years, growing emphasis has been given on the role of CD4 T cells for mediating effective antitumor responses [39, 40]. Supporting this notion, neoantigen-targeting therapies require the presence of both MHC class I and class II neoantigens to induce significant antitumor effects [41].

A limitation of our study is that the experiments were conducted within a specific allogeneic setting, and consequently the findings can be applicable solely to allogeneic environments. Nonetheless, the principle of inducing antitumor or antiviral acquired T cell immunity in a syngeneic context has been validated through previous studies in murine models [13, 35, 42]. In addition, our model did not monitor the long-term antitumor effect of the memory-phenotype T cells because mice ultimately died of graft-versus-host disease due to a xenogeneic immune reaction. However, proliferating memory-phenotype T cells at day 7 after the combination treatment included CD45RA^−^CCR7^+^ central memory T cells (Fig. S2F), indicating the potential for generating long-lived memory T cells after iNKT cell-targeted therapy. Likewise, increased central memory T cells were observed at day 7 after iNKT cell-targeted treatment before long-term memory formation in previous murine models [16, 36, 43].

In summary, our findings provide a proof of concept that αGalCer/APC-stimulated allogeneic iPSC-iNKT cells can effectively elicit antitumor T-cell immunity. This approach does not necessitate specific antigens and HLA matching while still facilitating diverse antitumor T-cell responses. The mechanism underlying this antitumor activity is personalized; however, the therapeutic methodology is applicable to a broad population because of its CD1d-restricted iNKT cell nature. Moreover, this therapy is not limited to a specific type of cancer but can be applied to various cancers.

## Supplementary Information

Below is the link to the electronic supplementary material.

Supplementary Material 1.

Supplementary Material 2.

Supplementary Material 3.

## Data Availability

Single-cell RNA-seq and TCR-seq data have been deposited at Gene Expression Omnibus as GSE292489.

## Abbreviations

αGalCer/APC: α-galactosylceramide-pulsed antigen-presenting cells
iNKT cells: Invariant natural killer T cells
iPSC-iNKT cells: Induced pluripotent stem cell-derived iNKT cells
CTV: CellTrace violet
CAR: Chimeric antigen receptor
DCs: Dendritic cells
DC/Gal: Dendritic cells pulsed with αGalCer
PBMC: Peripheral blood mononuclear cell
PDX: Patient-derived xenograft
TCR: T cell receptor
TILs: Tumor-infiltrating human lymphocytes
