# Supplementary material for: Preclinical efficacy of combination therapy with allogeneic induced pluripotent stem cell-derived invariant natural killer T and α-galactosylceramide-pulsed antigen-presenting cells
Source: Stem Cell Res Ther. 2026 Mar 29;17:150. doi: 10.1186/s13287-026-04994-7 (PMC13104297; doi:10.1186/s13287-026-04994-7)
Supplement: Supplementary file 1 — Supplementary Material 1. [file 13287_2026_4994_MOESM1_ESM.docx]

**MATERIALS AND METHODS**

**Reagents**

The reagents and resources used in this study are listed in Supplementary Table S2.

**Mice**

Human IL-7/15 (hIL-7/15) knock-in NSG mice (NSG.Cg-STOCK-Il7^tm1.1(IL7)HKO^ Il15^tm1.1(IL15)HKO^) were generated at RIKEN previously for iPSC-iNKT cells to survive in mice[1]. All mice were bred and maintained under specific pathogen-free conditions at the Jackson Laboratory Japan and the RIKEN animal facility. 4-5 week male hIL-7/15 NSG mice were delivered from the Jackson Laboratory Japan to RIKEN. Our study exclusively examined male mice for breeding reasons. Inhalation anesthesia with isoflurane (Viatris, Canonsburg, PA) was used during tumor transplantation. Mice were euthanized by cervical dislocation. This work has been reported in line with the ARRIVE guidelines 2.0. All animal experiments were performed under guidelines and approved by RIKEN's Institutional Animal Care and Use Committee.

**Patient-derived tumor and peripheral blood mononuclear cells (PBMCs)**

The patient-derived lung carcinoma, LC-06, was obtained from the Central Institute for Experimental Medicine and Life Science (Kanagawa, Japan). Human PBMCs were obtained from a healthy volunteer donor at RIEKN with written informed consent. All experiments were performed with authorization from the Institutional Review Board for Human Research at RIKEN Integrative Medical Sciences (IMS) and Chiba University.

**Human induced pluripotent stem cells**

The iNKT-iPSC clone was generated from a healthy donor-derived iNKT cell as previously described[2]. iPSCs were maintained in StemFit AK02N (Ajinomoto, Tokyo, Japan) on iMatrix-511 (Matrixome, Osaka, Japan).

**Generation of iPSC-iNKT cells**

iPSC-iNKT cells were generated from iNKT cell-derived iPSCs (iNKT-iPSCs) using the OP9/OP9DLL1 stromal cell co-culture system as previously described with a slight modification[2]. iNKT-iPSC colonies were mechanically divided and then seeded on mitomycin-C (Sigma-Aldrich, St. Louis, M)-treated OP9 overconfluent 10 cm dishes filled with 10 ml of OP9 medium, that is, MEMα (Life Technologies, Massachusetts, CA) with 20% FBS (fetal bovine serum, Gibco, Massachusetts, CA) supplemented with 10 μM Y-27632, (FUJIFILM Wako Pure Chemical, Osaka, Japan). The next day and on day 7, the medium was replaced with 10 ml of fresh medium. On day 13, progenitor cells were harvested. To remove stromal cells and aggregated cells, cells were passed through an EASYstrainer (100 μm, Greiner Bio-One, Frickenhausen, Germany). Then, the cells were plated on a mitomycin-C-treated OP9/DLL1 semi-confluent dish in OP9 medium containing 5 ng/ml of hIL-7(R&D systems, Minneapolis, MN), 5ng/ml of hFlt-3L (R&D), and 10 ng/ml of hSCF (R&D). On day 16 and day 23, semi-adherent cells were collected and passaged into a new dish layered with mitomycin C-treated OP9/DLL1 cells. On day 30, immature iPSC-iNKT cells were collected and frozen. Thawed immature iPSC-iNKT cells were cultured in MEMα with 20% hABserum (human Male AB serum, Access Biologicals, Vista, CA) with 5 ng/ml of hIL-7 and 10 ng/ml of hIL-15 (Peprotech, Cranbury, NJ) for 9 to 12 days using a FlexiRoll Cell Roller (5 rpm, Argos Technologies, Vernon Hills, IL).

**Murine bone marrow-derived dendritic cells**

We generated αGalCer-pulsed murine bone marrow-derived dendritic cells (DC/Gal) as previously described[3] with a slight modification. Briefly, we collected bone marrow cells from C57BL/6 mice and cultured them in RPMI 1640 medium (Thermo Fisher Scientific) supplemented with 10% FBS (Gibco) and 50 ng/ml of murine GM-CSF (R&D). To mature the DCs, we added 100 ng/ml of αGalCer (Funakoshi, Tokyo, Japan) on day 7 and 100 ng/ml of LPS (InvivoGen, San Diego, CA) on day 8. Mature αGalCer-pulsed DCs were collected on day 9.

**In vivo cytotoxicity Assay**

To evaluate the adjuvant activity of iPSC-iNKT cells stimulated by αGalCer/APC, we established a human PBMC-transplanted patient-derived xenograft (PDX) model. We inoculated ten blocks of frozen patient-derived lung cancer (LC-06) subcutaneously in 6-8 week male hIL-7/15 knock-in NSG mice on day -8 and transplanted 2 x 10^6^ human PBMCs into the tumor on day 0. Mice were allocated so that tumor size was balanced at the start of treatment. We separately injected 3 x 10^6^ iPSC-iNKT cells and/or 1 x 10^6^ DC/Gal cells into the tumor on day 1 and measured the tumor size in three directions. Tumor volume was determined using a caliper and applying the formula (volume = 0.52 × [long diameter] × [short diameter] × [height]) cm^3^. The examiner was blinded to the treatment group. We excluded the mice that died before day 21.

**Hematoxylin & eosin (H&E) staining**

Removed tumors were fixed in 4% Paraformaldehyde phosphate buffer Solution (Fujifilm Wako, Osaka, Japan), and embedded in paraffin. Tissues were sectioned (5mm thickness) and slides were stained with hematoxylin and eosin by Biopathology Institute Co., Ltd (Oita, Japan). The mounted slides were then examined and photographed using an Olympus DP27 microscope (Tokyo, Japan).

**Tumor-infiltrating lymphocyte (TIL) analysis**

For isolation of TILs, we digested LC-06 with collagenase D (Roche, Basel, Switzerland). Human TILs were obtained using human CD45 MicroBeads (Miltenyi, Bergisch Gladbach, Germany) according to the manufacturer’s protocol. To identify the immune cells in TILs, we used PE-labeled mCD45, Krome Orange-labeled hCD45, BV421-labeled CD3, FITC-labeled Va24, PE-Cy7-labeled CD56, and7-Amino-Actinomycin D (7AAD). To identify expanded memory-phenotype T cells, we used APC-labeled mCD45, Krome Orange-labeled hCD45, BV421-labeled CD3, FITC-labeled Va24, PE-labeled CCR7, PE-Cy7-labeled CD45RA, BV510-labeled CD8, APC-Cy7-labeld CD4, BV711-labeled CD27, and 7AAD. These compounds were incubated with TILs in 100 μl of PBS for 20 min in a refrigerator. Detailed antibody information is listed in Supplementary Table S2. Flow cytometric data were acquired with a FACS Canto II or a FACS Aria III instrument (BD Biosciences, Franklin Lakes, NJ) running FACSDiva and analyzed with Flowjo software (BD Biosciences).

**scRNA-Seq and scVDJ-Seq analysis**

We performed single-cell analysis with TILs from eight mice from 4 groups and pre-injected PBMCs. The collagenase D-digested tumor samples were stored in liquid nitrogen. The samples were thawed, and human CD45-positive cells were collected by a FACS Aria III device (BD Biosciences). Cells were encapsulated in droplets by the Chromium Controller (10x Genomics, Pleasanton, CA). The target cell number was 10,000 per mouse. The scRNA-Seq libraries were prepared using Chromium Next GEM Single Cell 5’ Reagent Kits (v2) according to the manufacturer’s protocol (10x Genomics). T-cell receptor (TCR) V(D)J segments (scVDJ-Seq) were enriched from amplified complementary DNA derived from 5’ libraries via PCR amplification using a Chromium Single Cell Human TCR Amplification Kit for Human T Cells according to the manufacturer’s protocol (10x Genomics). The generated scRNA-Seq and scVDJ-Seq libraries were sequenced on a DNBSEQ-G400RS (MGI, Shenzhen, China) with paired-end dual-indexing (26 cycles Read 1, 10 cycles i7, 10 cycles i5, 90 cycles Read 2). Sequence reads from all samples were processed and scRNA-Seq data were aggregated using the Cell Ranger (v6.1.2) pipeline with refdata-gex-GRCh38-2020-A (10x Genomics) as reference. Next, scVDJ-Seq data were aggregated using the Cell Ranger (v6.1.2) ‘vdj’ pipeline with refdata-cellranger-vdj-GRCh38-alts-ensembl-5.0.0 (10x Genomics) as reference. The integration process of scRNA-Seq and scVDJ-Seq in the same cell was performed using the ‘cellranger’ multi-pipeline. These procedures were performed at the Kazusa DNA Research Institute (Chiba, Japan). Aggregated data were further analyzed using Seurat v4 (<https://satijalab.org/seurat/>). We included cells whose percentage of mitochondrial genes was below 5 and whose number of genes detected per cell was above 1000. Principal component analysis (PCA) was performed using RunPCA (number of calculated principal components (PC) was 100), and enrichment of each PC was calculated using the JackStraw and ScoreJackStraw function (num.replicate = 100). PCs that were significantly enriched (P ≤ 0.05) were selected for clustering and dimensional reduction analysis. We visualized single-cell gene expression as UMAP, dot plots, and bar plots. Clonotype size was determined by counting the number of overlapping amino acid sequences in the antigen-recognizing CDR3 region. The counts were categorized into five groups: Extra large (100 < X), Large (50 < X ≤ 100), Medium (20 < X ≤ 50), Small (1 < X ≤ 20), and Single (0 < X ≤ 1).

**Cell lines**

We purchased Jurkat cells (InvivoGen, San Diego, CA) and generated TCRα/β-KO Jurkat cells. We knocked out the T cell receptor alpha constant (*TRAC*) and T cell receptor beta constant (*TRBC*) genes by the CRISPR/Cas9 system as previously described[4]. The sequences of the used crRNA were as follows: *TRAC*, 5ʹ-TGTGCTAGACATGAGGTCTA-3ʹ; and *TRBC1*, 5ʹ-GCAGTATCTGGAGTCATTGA-3ʹ. The *TRAC*-KO was performed first and confirmed by the negativity of TCR expression using an anti-TCRα/β antibody (BioLegend, San Diego, CA). *TRBC1* was then knocked out in the TRAC-KO Jurkat cells. *TRBC*-KO was confirmed in the identified TCR-KI TCRα/β-KO Jurkat cells by the negativity of TCRβ8 expression (Beckman Coulter, Brea, CA), which is the original TCRβ of Jurkat cells.

**Tumor specificity assay**

Identified TCRs were transfected with linearized expression plasmids into TCRα/β-KO Jurkat cells. The expression plasmids, pRP[Exp]-EF1A>TCRb:P2A:TCRa:T2A:Puro, were constructed by VectorBuilder Inc. (Chicago, IL). The vector IDs are VB240314-1760jrq, VB240314-1761sut, VB240314-1757cae, VB240314-1754vtx, which can be used to retrieve detailed information about the vector on vectorbuilder.com. The plasmids were cut by the PvuI restriction enzyme (R0150, New England Biolabs, Ipswich, MA) and purified with NucleoSpin Gel and PCR Clean-up extraction Kit (Macherey-Nagel, Germany). They were transfected using the NEON transfection system (Invitrogen). Plasmid-inserted and TCR-transfected Jurkat cells were selected using puromycin. TCR-transfected Jurkat cells were incubated in an incubator for 22-24 hours with collagenase D-digested LC-06 or bone marrow cells of hIL-7/15 NSG mice and assessed by CD69 expression for their reactivity to target cells.

**Depletion of human CCR7^+^ cells in tumor-bearing mice**

We administered 25 μg of anti-human CCR7 (clone 150503; R&D Systems, Minneapolis, MI, USA) or isotype control (clone 20102; R&D Systems, Minneapolis, MI, USA) antibody into LC-06 tumors 7 days after the combination therapy. To confirm that human CCR7^+^ cells were depleted by anti-human CCR7 antibody, TILs were collected two days after human PBMCs and antibody administration. Isolated TILs from LC-06 tumors were analyzed by flow cytometry using PE-labeled mCD45, Krome orange-labeled hCD45, APC-Cy7-labeled CD3, PE-Cy7-labeled CCR7 (clone G043H7, BioLegend, San Diego, CA, USA), APC-labeled CD62L, and 7AAD. These reagents were incubated with TILs in 100 μl of PBS for 20 min in a refrigerator. Detailed antibody information is listed in Supplementary Table S2. Flow cytometric data were acquired on a FACS Canto II instrument (BD Biosciences) using FACSDiva and analyzed with the FlowJo software (BD Biosciences).

**Statistical analysis**

Statistical analyses were performed using GraphPad Prism. Differences were analyzed using unpaired t-test. P<0.05 was considered statistically significant.

**Reference**

1. Matsuda M, Ono R, Iyoda T, Endo T, Iwasaki M, Tomizawa-Murasawa M, et al. Human NK cell development in hIL-7 and hIL-15 knockin NOD/SCID/IL2rgKO mice. Life Sci Alliance. 2019;2. doi:10.26508/lsa.201800195

2. Yamada D, Iyoda T, Vizcardo R, Shimizu K, Sato Y, Endo TA, et al. Efficient Regeneration of Human Vα24(+) Invariant Natural Killer T Cells and Their Anti-Tumor Activity In Vivo. Stem Cells. 2016;34:2852-60. doi:10.1002/stem.2465

3. Fujii S, Shimizu K, Kronenberg M, Steinman RM. Prolonged IFN-gamma-producing NKT response induced with alpha-galactosylceramide-loaded DCs. Nat Immunol. 2002;3:867-74. doi:10.1038/ni827

4. Aoki T, Takami M, Takatani T, Motoyoshi K, Ishii A, Hara A, et al. Activated invariant natural killer T cells directly recognize leukemia cells in a CD1d-independent manner. Cancer science. 2020;111:2223-33. doi:10.1111/cas.14428
