# Supplementary material for: Preclinical efficacy of combination therapy with allogeneic induced pluripotent stem cell-derived invariant natural killer T and α-galactosylceramide-pulsed antigen-presenting cells
Source: Stem Cell Res Ther. 2026 Mar 29;17:150. doi: 10.1186/s13287-026-04994-7 (PMC13104297; doi:10.1186/s13287-026-04994-7)
Supplement: Supplementary file 2 — Supplementary Material 2. [file 13287_2026_4994_MOESM2_ESM.docx]

**
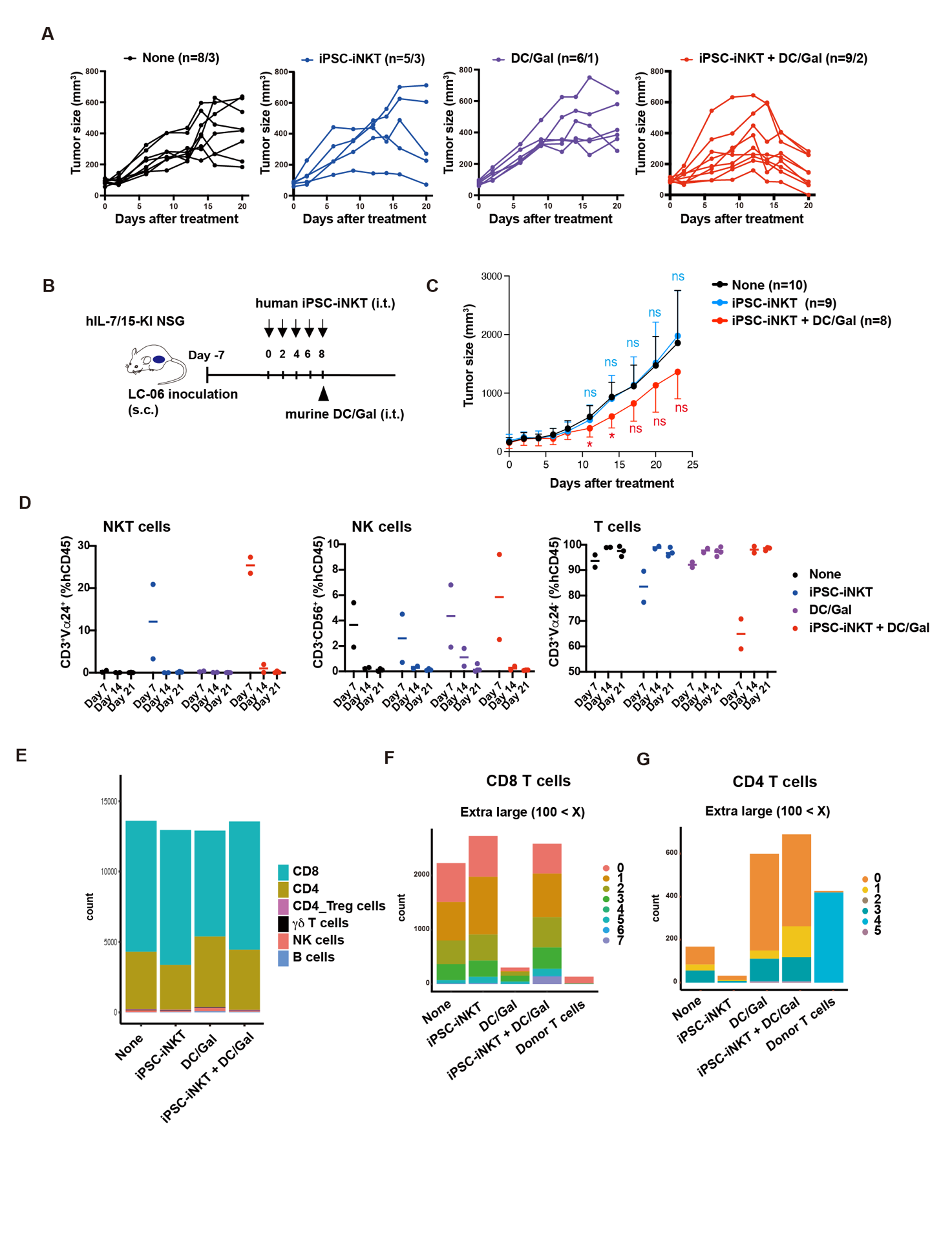
**

**Supplementary Figure S1.**

(A) Tumor size in each mouse in each treatment group. The number of mice in each experimental cohort is indicated with those excluded due to death before the end of the study. (B) Experimental scheme of PDX model without human PBMC. LC-06 was transplanted into human IL-7/15 knock-in NSG at day -7. iPSC-iNKT cells were administered from day 0 to 8 every other day. Murine DC/Gal was injected intratumorally on day 8. Tumor size was measured until day 23. (C) The tumor size in each treatment group (None: n=10, iPSC-iNKT: n=9, and iPSC-iNKT+DC/Gal: n=8). Data represent mean ± SEM. Statistical analyses were performed between none and iPSC-iNKT or iPSC-iNKT + DC/Gal at each time point. *, P < 0.05 (unpaired t-test); ns, not significant. (D) Human immune cell ratio in TILs at each time point in each treatment group. (E) Cell type distribution of day 14 human CD45^+^ TILs analyzed by scRNA-seq. (F) Bar plot showing clusters of CD8 T cells with extra-large TCR clone size. (G) Bar plot showing clusters of CD4 T cells with extra-large TCR clone size.


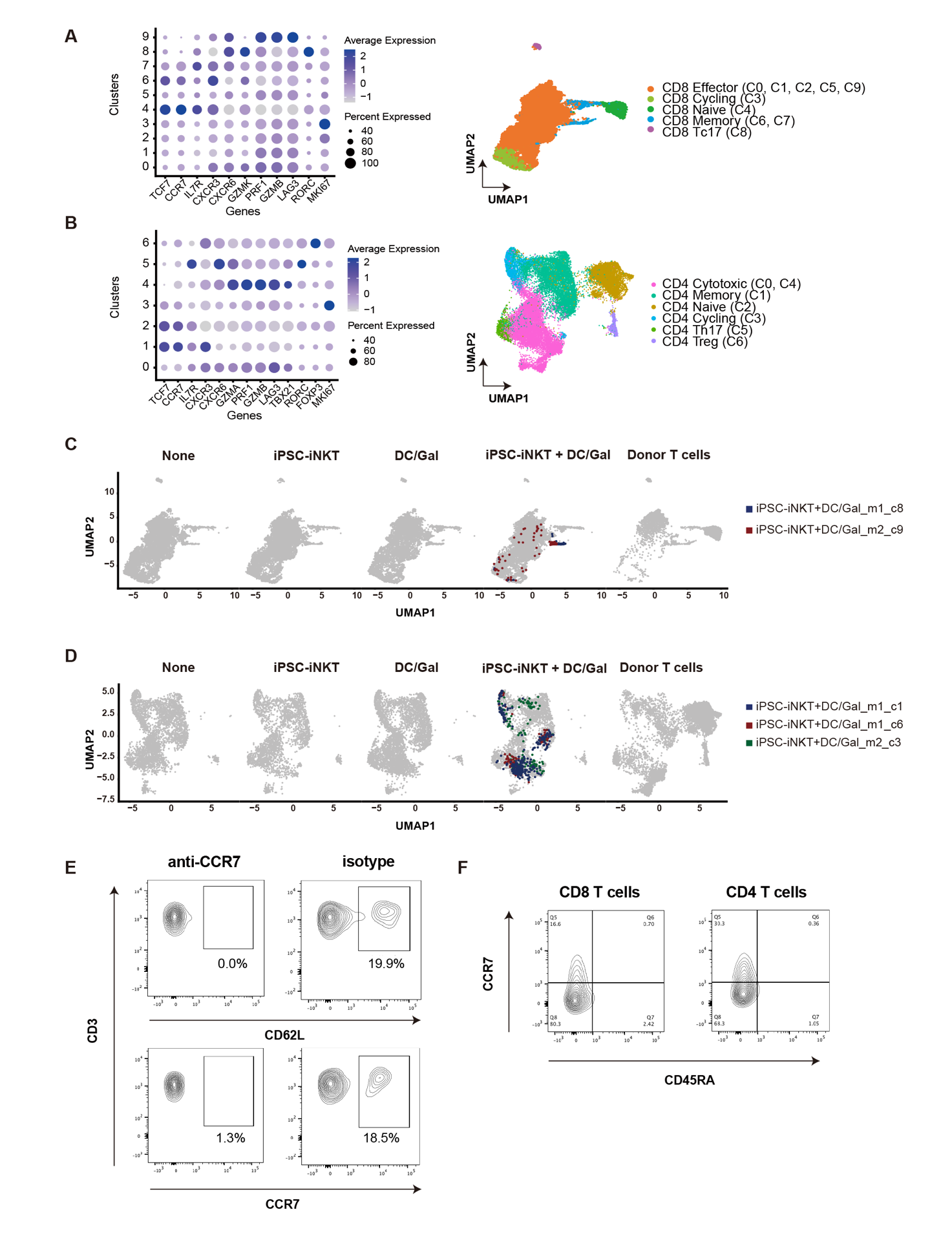


**Supplementary Figure S2.**

(A) Dot plot of CD8 T cell clusters (left) and UMAP of CD8 T cells categorized by phenotypes (right). Annotations were assigned based on feature gene expression shown in the left dot plot. (B) Dot plot of CD4 T cell clusters (left) and UMAP of CD4 T cells categorized by phenotypes (right). Annotations were assigned based on feature gene expression shown in the left dot plot. (C) Cells with identified clonotypes in CD8 T cells across all treatment groups. (D) Cells with identified clonotypes in CD4 T cells across all treatment groups. (E) Depletion of human CCR7^+^ T cells by anti-CCR7 antibody. Representative flow cytometry plots for expression of CD62L and CCR7 of human TILs (human CD3^+^/CD45^+^/7AAD^-^ cells) at 2 days after injection of anti-CCR7 (clone 150503; R&D Systems, Minneapolis, MN, USA) or isotype control (clone 20102; R&D Systems) antibody into LC-06 tumors are shown. APC-labeled anti-CD62L antibody (clone SK11, BD Bioscience, San Jose, CA, USA) and PE-Cy7-labeled anti-CCR7 antibody (clone G043H7, BioLegend, San Diego, CA, USA) were used for the detection of human CD62L^+^/CCR7^+^ T cells. (F) Presence of CD45RA^-^CCR7^+^ central memory T cells among CTV^low^ T cells. CTV^low^ T cells in Fig. 4A were further analyzed for the expression of CD45RA.
