# Supplementary material for: Preclinical efficacy of combination therapy with allogeneic induced pluripotent stem cell-derived invariant natural killer T and α-galactosylceramide-pulsed antigen-presenting cells
Source: Stem Cell Res Ther. 2026 Mar 29;17:150. doi: 10.1186/s13287-026-04994-7 (PMC13104297; doi:10.1186/s13287-026-04994-7)
Supplement: Supplementary file 3 — Supplementary Material 3. [file 13287_2026_4994_MOESM3_ESM.docx]

| CD8 TCR clonotypes | TRA_v_gene | TRA_j_gene | TRA_c_gene | TRA_cdr3 | TRB_v_gene | TRB_j_gene | TRB_c_gene | TRB_cdr3 |
| --- | --- | --- | --- | --- | --- | --- | --- | --- |
| iPS-NKT+DC/Gal_m1_c8 | TRAV6 | TRAJ13 | TRAC | CAPPSGGYQKVTF | TRBV7-8 | TRBJ2-2 | TRBC2 | CASSFRTGGLGTGELFF |
| iPS-NKT+DC/Gal_m2_c9 | TRAV8-2 | TRAJ6 | TRAC | CVVSDWGGGSYIPTF | TRBV7-8 | TRBJ1-2 | TRBC1 | CAGHLGGADGYTF |
| iPS-NKT_m2_c1 | TRAV1-1 | TRAJ30 | TRAC | CAVEENRDDKIIF | TRBV7-6 | TRBJ2-1 | TRBC2 | CAAGENNEQFF |
| None_m1_c3 | TRAV17 | TRAJ48 | TRAC | CAKRGAISNFGNEKLTF | TRBV7-6 | TRBJ1-1 | TRBC1 | CASSLDLREPAFF |
| CD4 TCR clonotypes | TRA_v_gene | TRA_j_gene | TRA_c_gene | TRA_cdr3 | TRB_v_gene | TRB_j_gene | TRB_c_gene | TRB_cdr3 |
| iPS-NKT+DC/Gal_m1_c1 | TRAV8-3 | TRAJ39 | TRAC | CAVAPRGAGNMLTF | TRBV2 | TRBJ2-2 | TRBC2 | CAGGQLSIGELFF |
| iPS-NKT+DC/Gal_m1_c6 | TRAV22 | TRAJ17 | TRAC | CAVGAAGNKLTF | TRBV29-1 | TRBJ2-7 | TRBC2 | CSVEDNRGHTYEQYF |
| iPS-NKT+DC/Gal_m2_c3 | TRAV38-1 | TRAJ58 | TRAC | CAFAETSGSRLTF | TRBV30 | TRBJ1-5 | TRBC1 | CAWTGQFNQPQHF |
| None_m1_c6 | TRAV12-2 | TRAJ20 | TRAC | CAVIGSNDYKLSF | TRBV6-1 | TRBJ1-4 | TRBC1 | CASSDSRGDEKLFF |
| DC/Gal_m2_c1 | TRAV12-1 | TRAJ3 | TRAC | CASPRASKIIF | TRBV5-1 | TRBJ2-2 | TRBC2 | CASSLGGVGDTGELFF |
| iPS-NKT+DC/Gal_m1_c4 | N/A | N/A | N/A | N/A | TRBV2 | TRBJ2-2 | TRBC2 | CAGGQLSIGELFF |
| DC/Gal_m2_c3 | TRAV41 | TRAJ45 | TRAC | CAVRRSGGGADGLTF | TRBV16 | TRBJ1-2 | TRBC1 | CASSQSLGQGAYGYTF |
| DC/Gal_m2_c2 | TRAV13-2 | TRAJ39 | TRAC | CAENNAGNMLTF | TRBV6-5 | TRBJ2-7 | TRBC2 | CASSKDREGYEQYF |

Supplementary Table S1. TCR clonotypes in Figure 3A

Supplementary Table S2. Reagents and resources

| **REAGENT or RESOURCE** | **SOURCE** | **IDENTIFIER** |
| --- | --- | --- |
| Antibodies | | |
| Anti-human Va24-FITC | Beckman Coulter | Cat#IM1589 |
| Anti-human CD3-BV421 | BioLegend | Cat#300434; RRID: AB_10962690 |
| Anti-human CD56-PE-Cy7 | BioLegend | Cat#362510; RRID: AB_2563927 |
| Anti-human CD45-Krome Orange | Beckman Coulter | Cat#B36294; RRID: AB_2833027 |
| Anti-mouse CD45-PE | BioLegend | Cat#103106; RRID: AB_312971 |
| Anti-human CCR7-PE | BioLegend | Cat#353204; RRID: AB_10913813 |
| Anti-human CD45RA-PE-Cy7 | BioLegend | Cat#304126; RRID: AB_10708879 |
| Anti-human CD8-BV510 | BioLegend | Cat#344732; RRID: AB_2564624 |
| Anti-human CD3-BV605 | BioLegend | Cat#317322; RRID: AB_2561911 |
| Anti-human CD27-BV711 | BioLegend | Cat#302834; RRID: AB_11219201 |
| Anti-human CD4-APC-Cy7 | BD Pharmingen | Cat#566913; RRID: AB_2739681 |
| Anti-mouse CD45-APC | BioLegend | Cat#103112; RRID: AB_312977 |
| Anti-human CD3-APC | BioLegend | Cat#300412; RRID AB_314066 |
| Anti-human CD69-FITC | BD Pharmingen | Cat#555530; RRID AB_395915 |
| Purified mouse anti-human CCR7 antibody | R&D | Cat#MAB197; RRID: AB_2072803 |
| Mouse IgG 2A isotype control | R&D | Cat#MAB003; RRID: AB_357345 |
| Anti-human CCR7-PE-Cy7 | BioLegend | Cat#353226; RRID: AB_11126145 |
| Anti-human CD3-APC-Cy7 | BioLegend | Cat#344818; RRID: AB_10645474 |
| Anti-human CD62L-APC | BD Pharmingen | Cat#566791; RRID: AB_2869869 |
| Anti-human TCRα/β-PE | BioLegend | Cat#306708; RRID AB_314646 |
| Anti-human TCRβ8-FITC | Beckman Coulter | Cat#IM1233; RRID: AB_130922 |
| Biological samples | | |
| iNKT-iPSCs, hi133-4 | Yamada et al.^2^ | N/A |
| OP9 | Yamada et al.^2^ | N/A |
| OP9DLL1 | Yamada et al.^2^ | N/A |
| Healthy donor PBMCs | RIKEN | N/A |
| Patient-derived xenografts (PDX), LC-06 | Central Institute for Experimental Medicine and Life Science | N/A |
| Chemicals, peptides, and recombinant proteins | | |
| StemFit AK02N | Ajinomoto | Cat#AK02N |
| iMatrix-511 | Matrixome | Cat#892012 |
| mitomycin-C | Sigma-Aldrich | Cat#M4287-2MG |
| MEMα，nucleosides, powder | Life Technologies | Cat#11900-073 |
| Human IL-7 | R&D | Cat#207-IL-025 |
| Y-27632 | Fujifilm Wako | Cat#036-24023 |
| Human Flt-3L | R&D | Cat#308-FK-025 |
| Human SCF | R&D | Cat#255-SC-050 |
| Human IL-15 | Peprotech | Cat#PEP-200-15-10 |
| RPMI 1640 | Themo Fisher Scientific | Cat#11875093 |
| Murine GM-CSF | R&D | Cat#415-ML |
| LPS | InvivoGen | Cat#tlrl-eklps |
| αGalCer | Funakoshi | Cat#KRN7000 |
| 4% Paraformaldehyde phosphate buffer Solution | Fujifilm Wako | Cat#163-20145 |
| Human CD45 microbeads | Miltenyi Biotech | Cat#130-045-801 |
| PvuI restriction enzyme | New England Biolabs | Cat#R0150 |
| Alt-R^®^ S.p. dCas9 Protein V3 | Integrated DNA Technologies | Cat#1081067 |
| Alt-R^®^ Cas9 Electroporation Enhancer | Integrated DNA Technologies | Cat#1075916 |
| collagenase D | Roche | Cat#11088858001 |
| Isoflurane | Viatris | VTRS |
| Commercial kits | | |
| CellTrace™ Violet Cell Proliferation Kit | Themo Fisher Scientific | Cat#C34557 |
| Chromium Next GEM Single Cell 5ʹ Reagent Kit v2 | 10X Genomics | PN-1000263 |
| Chromium Next GEM Chip K Single Cell Kit | 10X Genomics | PN-100028 |
| Chromium Single Cell Human TCR Amplification Kit | 10X Genomics | PN-1000252 |
| Library Construction Kit | 10X Genomics | PN-1000190 |
| Dual Index Kit TT Set A | 10X Genomics | PN- 1000215 |
| Deposited data | | |
| Raw and analyzed data: scRNA-seq, scTCR-seq | This paper | GEO: GSE292489 |
| Experimental models: Cell lines | | |
| Human: Jurkat cells | InvivoGen | Cat#jktl-nfat |
| Experimental models: Organisms/strains | | |
| Mouse: hIL-7×15 NSG mice (NSG.Cg-STOCK-Il7^tm1.1(IL7)HKO^ Il15^tm1.1(IL15)HKO^) | Matsuda et al.^1^ | N/A |
| Mouse:C57BL/6J | Jackson laboratory Japan | Strain#000664 |
| Oligonucleotides | | |
| crRNA targeting sequence: TRAC, TGTGCTAGACATGAGGTCTA | Aoki et al. ^4^ | N/A |
| crRNA targeting sequence: TRBC, GCAGTATCTGGAGTCATTGA | Aoki et al.^4^ | N/A |
| Alt-R^®^ CRISPR-Cas9 tracrRNA | Integrated DNA Technologies | Cat#1072533 |
| Recombinant DNA | | |
| Plasmid: pRP[Exp]-EF1A>[CD4_TCRb_1]:P2A;[CD4_TCRa_1]:T2A:Puro | VectorBuilder | VB240314-1760jrq |
| Plasmid: pRP[Exp]-EF1A>[CD4_TCRb_2]:P2A:[CD4_TCRa_2]:T2A:Puro | VectorBuilder | VB240314-1761sut |
| Plasmid: pRP[Exp]-EF1A>[CD8_TCRb_1]:P2A:[CD8_TCRa_1]:T2A:Puro | VectorBuilder | VB240314-1757cae |
| Plasmid: pRP[Exp]-EF1A>[CD8_TCRb_2]:P2A:[CD8_TCRa_2]:T2A:Puro | VectorBuilder | VB240314-1754vtx |
| Software and codes | | |
| Flowjo v10 | BD | https://www.flowjo.com/ |
| Prism 9 | GraphPad | https://www.graphpad.com/ |
| Cell Ranger v6.1.2 | 10x genomics | https://www.10xgenomics.com/jp/support/software/cell-ranger/ |
| R v4.1.0 | CRAN | https://www.r-project.org |
| Seurat v4 | Satija et al. | https://satijalab.org/seurat/ |
| Matrix (version 1.5.1) | CRAN | https://cran.r-project.org/web/packages/Matrix/index.html |
| loomR (version 0.2.1.9000) | GitHub | https://github.com/mojaveazure/loomR |
| dplyr (version 1.1.4) | CRAN | https://cran.r-project.org/web/packages/dplyr/index.html |
| Stringr (version 1.5.1) | CRAN | https://cran.r-project.org/web/packages/stringr/index.html |
| ggplot2 (version 3.4.4) | CRAN | https://cran.r-project.org/web/packages/ggplot2/index.html |
| cowplot (version 1.1.3) | CRAN | https://cran.r-project.org/web/packages/cowplot/index.html |
